# Physical and chemical characterization of experimental newly formulated polymer infiltrated lithium disilicate ceramic network versus polymer infiltrated feldspathic ceramic network (an in-vitro study)

**DOI:** 10.1186/s12903-025-06134-8

**Published:** 2025-06-05

**Authors:** Alaa Hussein, Moustafa Aboushlieb, Nour A. Habib

**Affiliations:** 1 Biomaterials Department, Faculty of Dentistry, October 6 University, 6th of October, Egypt; 2 Dental Biomaterials Department, Faculty of Dentistry, Alexandria University, Alexandria, Egypt; 3 Biomaterials Department, Faculty of Dentistry, Cairo University, Cairo, Egypt

**Keywords:** PICN, PILN, Polymer infiltrated lithium disilicate ceramics, Hybrid ceramics

## Abstract

**Background:**

Polymer infiltrated ceramic network (PICN) is a hybrid dental ceramic that mimics the properties of tooth structures. Unfortunately, commercially available PICN still present limitations such as low strength. Thus, the current study was conducted to prepare polymer infiltrated lithium disilicate ceramic network (PILN) and compare it with commercially available PICN regarding microstructure and biaxial flexural strength.

**Methodology:**

A fine powder of lithium disilicate was produced by grounding ^IPS^e.max CAD/CAM blocks. A porous lithium disilicate ceramic networks containing 25% (PILN-25) and 20% (PILN-20) porosity were produced by firing at 820^O^C and 830^O^C respectively. Polymer was infiltrated and polymerized to form a dense PILN. A total of 69 specimens were prepared and assigned into three groups (*n* = 23) according to the type of ceramic used in fabrication of the ceramic network. The comparable group was Enamic, while the intervention groups were (PILN-25) and (PILN-20). For two intervention groups, porosity and density were measured before and after polymer infiltration using helium pycnometer (*n* = 3 at each stage). SEM was used for microstructure analysis (*n* = 9) and One specimen was examined under FESEM for better visualization of the crystalline phases. Additionally, three specimens (*n* = 3) from each group were assigned for XRD testing and, finally, ten specimens for each group (*n* = 10) were subjected to biaxial flexural strength test. The statistical significance level was set at *p* ≤ 0.005.

**Results:**

There was a statistically significant difference in biaxial flexural strength, PILN-20 recording the highest significant strength followed by PILN-25 and Enamic. PILN-25 showed higher porosity% than PILN-20 and the porosity decreased after polymer infiltration. PILN-20 showed higher density than PILN-25 and density increased after polymer infiltration. XRD revealed the presence of lithium disilicate crystals in both PILN-25 and PILN-20. SEM revealed highly interlocked ceramic and polymeric networks. FESEM revealed the presence of spherical lithium disilicate crystals.

**Conclusion:**

PILN is a new type of hybrid ceramic material with enhanced mechanical properties.

**Clinical implication:**

PILN can be used as a promising CAD/CAM block for creating high strength high esthetics dental restorations.

**Supplementary Information:**

The online version contains supplementary material available at 10.1186/s12903-025-06134-8.

## Background

For many years, the most predictable aesthetic correction of anterior teeth has been achieved through three classes of indirect restorative materials: porcelain-fused-to-metal (PFM), indirect composite, and all-ceramic restorations [1]. Driven by an enhanced awareness of aesthetics and biocompatibility, dentistry has shifted towards metal-free, tooth-colored restorations, which currently includes two main groups ceramics and composites [2].

Composites blocks are notable for their lower hardness, brittleness, higher resilience, and better machinability [3]. Their ability to be milled to a very low thickness offers new possibilities for use as minimally invasive bonded restorations [4]. However, their clinical performance is still inferior to their counterpart ceramic restorations in terms of strength, color matching and anatomic shape restoration [5]. On the other hand, ceramic blocks show excellent biocompatibility, good wear resistance, and high aesthetic performance [6]. Unfortunately, they still present limitations such as brittleness and marginal chipping during milling [7].

With the intention of optimizing their performance by combining positive characteristics of both ceramics and composite resins into a single material, a hybrid ceramic known as polymer infiltrated ceramic network (PICN) material has been developed. Since the introduction of Vita Enamic (Vita, Bad Säckingen, Germany) in 2013, the concept of PICN has gained widespread acceptance in the dental restoration industry [5, 8, 9].

PICN composites are typically described in litrature as having a 70–85% inorganic phase and a 15–30 wt% organic phase [10]. Options for the inorganic phase include powders of feldspathic, zirconia, and sodium aluminum silicate [11–13]. While the organic phase may include resins such as UDMA (urethane dimethacrylate), Bis-GMA (bisphenol A-glycidyl methacrylate), TEGDMA (triethylenglycol dimethacrylate), and even poly (methyl methacrylate) [14, 15].

Unlike conventional ceramic CAD/CAM materials, PICN is a type of ceramic characterized by interconnected networks comprising both continuous ceramic and resin phases [16, 17]. Improved properties are observed after the integration of the brittle ceramic network with the ductile polymer network. This integration is also claimed to provide greater structural reliability through a crack-stop function, which occurs when a crack propagating through the polymer network halts due to the presence of ceramic phase [3].

The mechanical characteristics of commercial PICN have been the subject of several foundational investigations. Results show that the properties of PICN mimic those of tooth structure [18]. Coldea et al., for example, demonstrated that PICN's Vickers hardness and elastic modulus were intermediate between those of dentin and enamel [3]. Research also suggests that PICN’s fatigue resistance to mechanical and thermal aging, as well as its machinability, contributes to its biomimetic mechanical properties [4, 5]^.^ PICN is also claimed to offer superior machinability and edge stability during milling [8, 19].

Although the combination of the crystalline matrix and the polymeric material has a positive impact on mechanical properties, this polymer infiltrated ceramic network still presents a low elastic modulus ~ 16.4–28.1 GPa and flexural strength ~ 150 MPa compared to other ceramic systems [3]. Due to their low strength, PICN can only be used on a limited range of teeth, and is not considered appropriate for molars, as clinical fracture may occur. Since the ceramic network significantly influences PICN performance, clinical fractures may be explained by the presence of a feldspathic network in PICN [16].

Based on the previous comparative studies on various CAD/CAM materials, including feldspathic and lithium disilicate ceramics, the latter possesses superior mechanical properties [21–25]. To the best of our knowledge, no systematic comparative study addressing this issue has been published. In this study, we investigated the effect of replacing the feldspathic ceramic network with a lithium disilicate ceramic network on the properties of polymer-infiltrated ceramic network (PICN). The aim of this study was to prepare a polymer-infiltrated lithium disilicate ceramic network (PILN) and compare it with commercially available PICN in terms of microstructure and biaxial flexural strength. The null hypothesis was that replacing the feldspathic ceramic network in commercially available PICN with a lithium disilicate ceramic network would not improve the final PICN's properties.

## Materials and methods

### Preparation and sintering of lithium disilicate inorganic preform

**Fig. 1:**
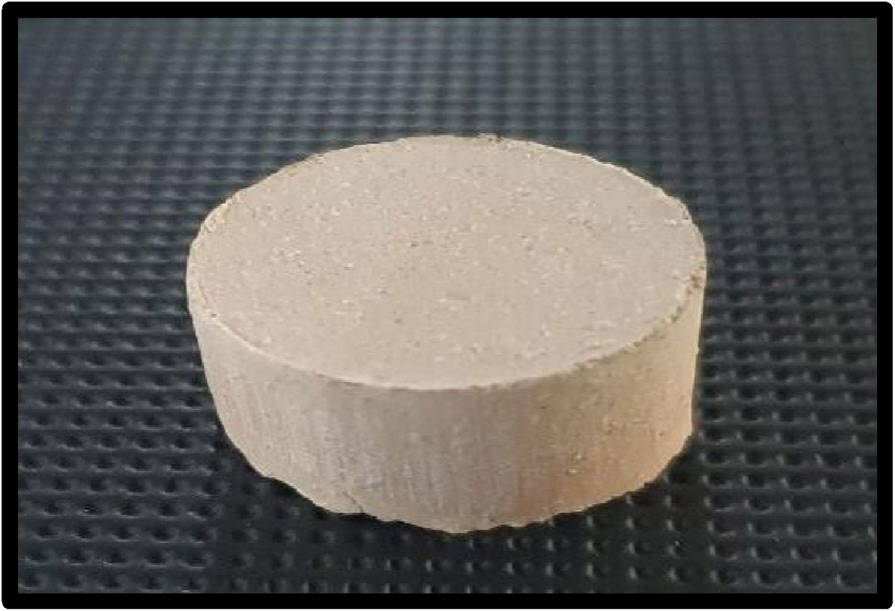
Porous ceramic preform before polymer infiltration

Based on pilot study, lithium disilicate ceramic powder was prepared from commercial blocks (Ivoclar vivadent AG FL-9494 Schaan/Liechtenstein, Switzerland) the powder was screened through 200-mesh sieve to obtain the lithium disilicate ceramic powder with an average diameter of 16 μm. Polyvinyl alcohol 3 wt.% solution (PVA) (MW = 22,000, OXFORD LAB FINE CHEM LIP, India) was used. Then, 0.75 g lithium disilicate ceramic powder was weighed and mixed with the PVA solution (~ 0.15 mL) to produce paste. The lithium disilicate paste was transferred to a stainless-steel mold (ϕ = 14 mm, h = 10 mm) and pressed at 6 MPa for 1 min to form green body preforms, which were then dried at 110ºC for 24 h (Fig. 1).

The preforms were then placed in an electric furnace, where the temperature was increased at a ramp rate of 90 °C/min from room temperature up to 550 °C and held for 2 h. Following this, the green body preforms were sintered for 1 h at two different temperatures: 820^O^C and 830^O^C. Four blocks were sintered at 820 ^O^C, and four blocks were sintered at 830 °C, with a heating rate of 30 °C/min. At the end of thermal treatment, the preforms were allowed to cool to ambient temperature using a cooling rate of 5◦C/min., resulting in porous lithium disilicate preforms. In the subsequent study, three groups were assigned: the control group consisting of polymer infiltrated feldspathic ceramic network (Enamic), group PILN-25 polymer infiltrated lithium disilicate ceramic network fired at 820 ^O^C and group PILN-20 polymer infiltrated lithium disilicate ceramic network fired at 830 ^O^C.

### Preparation of polymer-infiltrated lithium disilicate ceramics

A single component pre-hydrolyzed silane coupling agent (Bisco, Schaumburg il, USA) was dissolved in mixed ethanol/H2O (1:1 in volume). The porous lithium disilicate performers were then soaked in this solution and kept for 8 h at room temperature. The salinized preforms were vacuum dried under 60ºC and prepared for polymer infiltration. Next, the salinized performs were fully immersed in a monomer mixture of methyl methacrylate (MMA) combined with 0.5wt% benzoyl peroxide. Infiltration of methyl methacrylate (MMA) into the preforms was assisted by means of ultrasonic force, spin motive force and high centrifugal pressure.

**Fig. 2:**
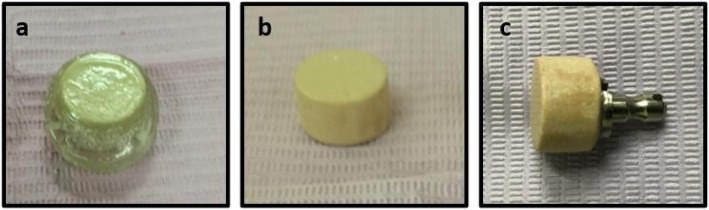
Polymer infiltrated lithium disilicate ceramic network block. **a** before finishing. **b** After finishing. **c** After cementation of metallic stub

High temperature thermal polymerization of the infiltrated preform was carried out by immersing it in a methyl methacrylate (MMA) solution within a water bath. The bath temperature was raised to 70 °C at a rate of 2 °C/min, and the process continued for 6 h to allow full polymerization. Following the main curing cycle, a post curing step was performed by holding the preform at 40 °C for an additional 2 h. The preform was then allowed to cool gradually, ensuring complete polymerization. Once the polymerization was finalized, any excess polymethyl methacrylate (PMMA) on the surface, along with any extraneous top and bottom layers, was removed. Finally, metallic stubs were attached to one end of each prepared block using cyanoacrylate glue to facilitate handling (Fig. 2).

### Sample size calculation

A sample size calculation is a crucial component of every research project to reduce the probability of error, adhere to ethical standards, specify the logistics of the study, and ultimately increase its success rates. Additionally, it is important for economic reasons: An undersized study can be a waste of resources due to its inability to yield valuable results, while an oversized one uses more resources than are necessary [62].The sample size calculation for this in-vitro study was done using the comparison of biaxial flexural strength of the prepared polymer infiltrated lithium disilicate ceramic network and polymer infiltrated felspathic ceramic network, as this was the primary outcome of our study. As reported in previous publication [7], the mean ± SD of flexure length in polymer infiltrated felspathic ceramic network was 137 ± 7 MPa. It was assumed that the prepared polymer infiltrated lithium disilicate ceramic network will increase flexural strength by at least 50%. Consequently, the minimum proper sample size was calculated, and 10 samples for each material were assigned to be able to reject the null hypothesis with 99% power at α = 0.05 level using Student’s t-test. Based on a previous study by [16, 26] using power 99% and significance level 0.05 using Student’s t test, 3 specimens were needed in each group for porosity, density, and microstructural characterization. Sample size calculation was performed using PS Power and Sample Size Calculations software, version 3.0.11 for MS Windows (William D. Dupont and Walton D., Vanderbilt University, Nashville, Tennessee, USA).

### Characterizations

#### Analysis of porosity and density

The porosity and the density of the ceramic preforms, prepared under different sintering temperatures, were quantified using the Gas Expansion Method. A helium pycnometer (UltraPyc 1200e, Model 2014, Quantachrome, USA), which utilizes 99.995% pure helium, was employed for these measurements. The Porosity of the PILN preforms was determined by measuring the pressure change of helium in a calibrated volume. The volume of the preform was measured at the room temperature, with an applied pressure of 131,000 Pa. Then, solid density and helium filled porosity of the preform were calculated using the following equations:

1 $$\rho g=\frac{{W}_{d}}{{V}_{g}}$$

Where:

ρg is the density, Wd is the dry weight and Vg is volume.

2 $$\varnothing=\frac{(\rho g-\rho b)\times100}{\rho g}$$

Where:

∅ is helium porosity, g is the solid density and b is the bulk density (with pores included).

To calculate density of the preform, bulk volume and dry weight of the preforms must be measured. The bulk volume and the dry weight of the preforms were estimated using direct methods; digital electronic balance 0.0001 g precision, and highly precise caliper (0.01 mm precision). Then the bulk density (b) was calculated using the following equation:

$$\rho b=\frac{Wd}{Vb}$$

Where:

vb is the bulk volume of the specimen and wd is the dry weight of the specimen.

Porosity and density measurements were again performed after polymer infiltration and proper curing following the same previously mentioned technique.

#### Determination of crystalline phases

X-ray diffraction (Shimadzu XRD-7000 diffractometer, shimadzu corporation, Tokyo, Japan) was also performed on three specimens from each group to visualize the crystal structure of lithium disilicate. Each specimen was scanned in bulk over the 2θ range of 5° to 90°, with 0.04°step size and 5-step intervals, and an angular resolution of 0.005° to identify crystalline phases. Three Enamic specimens were also examined to detect the presence or absence of crystalline phases. The captured XRD patterns were correlated with the patterns from the inorganic crystal structure database (COD) and analyzed.

#### Measurement of resin degree of conversion

The degree of conversion (DC) of C = C bonds in PMMA for groups PILN-25 and PILN-20 was determined using Raman Microscopy (300 HR-Evolution, Horiba Jobin Yvon; Stanmore, Middlesex, UK) with the following parameters: 50 mW He–Ne laser with 532 nm wavelength (≡ 2.33 eV), spectral resolution ~ 1 cm-1, spatial resolution ~ 1.5 μm, accumulation time 20 s, number of accumulations 1, and 100X magnification. A Raman shift range between 200 cm^−1^ and 3300 cm^−1^ was covered. Two characteristic bands 1648 cm − 1(stretching of methyl methacrylate C = C) and1736 cm − 1(stretching of carbonyl group C = O) were used to calculate the DC of polymerization. The ratios of absorbance intensities were calculated before and after polymerization using the following equation:

$$DC\%=1-\frac{R_{cured}}{\left(R_{cured}\right)\times100}$$

where R is the ratio of peak intensities at 1648 cm^−1 ^and 1736 cm^−^ bonds in cured and uncured methyl methacrylate respectively. The captured spectral peaks were correlated with those from database of PMMA Raman spectra and analyzed.

#### Microstructure and chemical structure analysis

For better visualization of the porosity in the PILN, a scanning electron microscope (LabSpec4.18 Horiba Jobin Yvon) was employed. Three representative specimens from group PILN-25 and group PILN-20 were examined before polymer infiltration. One representative specimen was examined using a high-resolution field emission scanning electron microscope (FESEM, Quanta FEG 250, equipped with Schottky field emission gun, Netherlands) to observe the grain shapes and sizes. After polymer infiltration, the specimens were re-examined, and three Enamic specimens were analyzed as received for comparison. Additionally, SEM analyses were performed on three representative polished specimens following surface-conditionings with 5%hydrofluoric acid to visualize of polymeric network after removal of ceramic network. The acid concentrations and conditioning times varied by material: Enamic specimens were etched for 60 s, while PILN-25 and PILN-20 specimens were etched for 20 s. Furthermore, three additional specimens were fired at 500 °C for 30 min to ensure visualization of the ceramic network after complete removal of the polymeric network.

#### Biaxial flexural strength measurement

A universal test instrument (Instron model 3345 universal testing machine. USA) was employed to measure the biaxial flexural strength of specimens. Ball on 3 balls method was applied, with a loading rate of 1 mm/min was used with a load cell of 5000 N, until failure. Ten polished disk-shaped specimens (12 mm in diameter) were prepared for each group by cutting from corresponding blocks according to ISO-6872, 2019 standard. Each disk specimen was placed on top of three stainless steel balls (each ball is 4 mm in diameter) (ø: 4 mm), arranged 120° apart on a circle with a 10 mm diameter (ø: 10 mm). A fourth ball (4-mm in diameter) (ø: 4 mm) was used to centrally load the specimen until fracture occurred. Biaxial flexural strength was calculated by using the following formula:

3 $$\sigma=\frac{-0.2387\;P\;(X-Y)}{d^2}$$

Where:

σ is the biaxial flexural strength (MPa), P the total load causing fracture (N), d is the specimen thickness at fracture origin (mm) and X and Y were determined as follow:

4 $$X=\left(1+v\right)in {\left(\frac{{r}_{2}}{{r}_{3}}\right)}^{2}+ \frac{\left(1-v\right)}{2} {\left(\frac{{r}_{2}}{{r}_{3}}\right)}^{2}$$

5 $$Y =\left(1+v\right) \left[1+in {\left(\frac{{r}_{1}}{{r}_{3}}\right)}^{2}\right]+ \left(1+v\right) {\left(\frac{{r}_{1}}{{r}_{3}}\right)}^{2}$$

Where:

v is Poisson’s ratio (values used in this study were 0.23 for Enamic group and 0.25 for PILN-25 and PILN-20 groups), r_1_ is the radius of support circle, r_2_ is the radius of loaded area, r_3_ is the radius of specimen and d is the specimen thickness at fracture origin.

### Statistical analysis

Numerical data were assessed for normality by examining the data distribution and calculating mean and median values using Prism 5 for windows version 5.01(GraphPad software Inc., La Jolla, CA, USA). Flexural strength data showed parametric distribution. Two-way ANOVA followed by Tukey test as post-Hoc test was used to analyze the flexural strength of different polymer infiltrated ceramic networks. Multiple comparisons were also conducted using analysis of variance (ANOVA). Data were represented by mean and standard deviation (SD) values. Differences between any two groups were considered as significant for *p ≤ 0.05.

## Results

### Porosity and density results

The results of porosity, as shown in (Table 1) showed that before polymer infiltration, the mean porosity percentage in PILN-25 was (25.02%), while in PILN-20, it was (19.87%). After polymer infiltration, the porosity decreased; the mean value of porosity percentage recorded by PILN-25 was (1.413%), while PILN-20 recorded (1.203%).

**Table 1:** The mean, standard deviation (SD) and p-value of the helium pycnometer porosity% of the two groups: PILN-25 and PILN-20 (*n* = 3)

Porosity by helium pycnometer %				
Group	PILN-25		PILN-20	
	Mean	SD	Mean	SD
Before infiltration	25.02^Aa^	0.08327	19.87%^Ab^	0.1504
After infiltration	1.413^Ba^	0.01528	1.203%^Ba^	0.01528
*P* value	*P* < 0.001*			

Superscripts with different capital letters indicate statistically significance difference within the same column

Superscripts with different small letters indicate statistically significance difference within the same row. Significance level *p* ≤ 0.001, *significant. # (*n* = 3)

Results of density measurement, as illustrated in (Table 2) showed that before polymer infiltration, the mean density of the specimens in PILN-25 was (1.863 g/cm^3^), while in PILN-20, the mean density was (1.970 g/cm^3^). After polymer infiltration, the density increased; the mean density recorded by PILN-25 was (2.533 g/cm^3^), while PILN-20 recorded (2.663 g/cm^3^). According to the literature, the density of Enamic is (2.09 g/cm^3^) [5].

**Table 2:** The mean, standard deviation (SD) and p-value of the Helium pycnometer density g/cm3 of the two groups: PILN-25 and PILN-20 (*n* = 3)

Density by helium pycnometer g/cm^3^				
Group	PILN-25		PILN-20	
	Mean	SD	Mean	SD
Before infiltration	1.863^Aa^	0.02082	1.970^Aa^	0.02646
After infiltration	2.533^Ba^	0.03055	2.663^Bb^	0.005773
*P* value	*P* < 0.001*			

Superscripts with different capital letters indicate statistically significance difference within the same column. Superscripts with different small letters indicate statistically significance difference within the same row

Significance level *p* ≤ 0.001

*significant. # (*n* = 3)

### Results of X-ray diffraction analysis

**Fig. 3:**
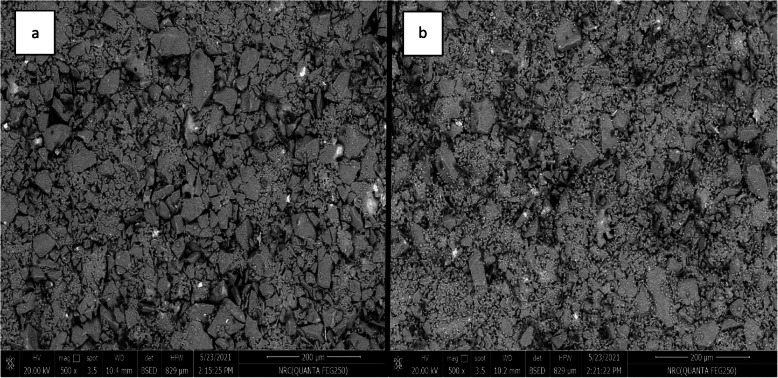
SEM of the ceramic preform showing apparent porosity (**a**) PILN-25 (**b**) PILN-20. both specimens show apparent porosity. Magnification 500x

The X-ray diffraction analysis of Enamic specimens displayed no dominant peaks, only a broad hump indicating an amorphous material without crystalline phases (Fig. 3a). The diffraction patterns of PILN-25 and PILN-20, however, demonstrated sharp, well-defined peaks, indicating that both materials had predominantly crystalline structure with very few broad bands which corresponded to the amorphous glassy phase (Fig. 3b &c). The dominant peaks were attributed to cristobalite and lithium disilicate crystals (Li_2_Si_2_O_5_) COD no. 96–231–0669 and COD no. 96- 231–0436. PILN-20 showed a higher amount of lithium disilicate crystals due to the higher sintering temperature of 830 ^O^C, as the increased thermal energy promoted the formation of more lithium disilicates.

### Results of Raman spectroscopy

**Fig. 4:**
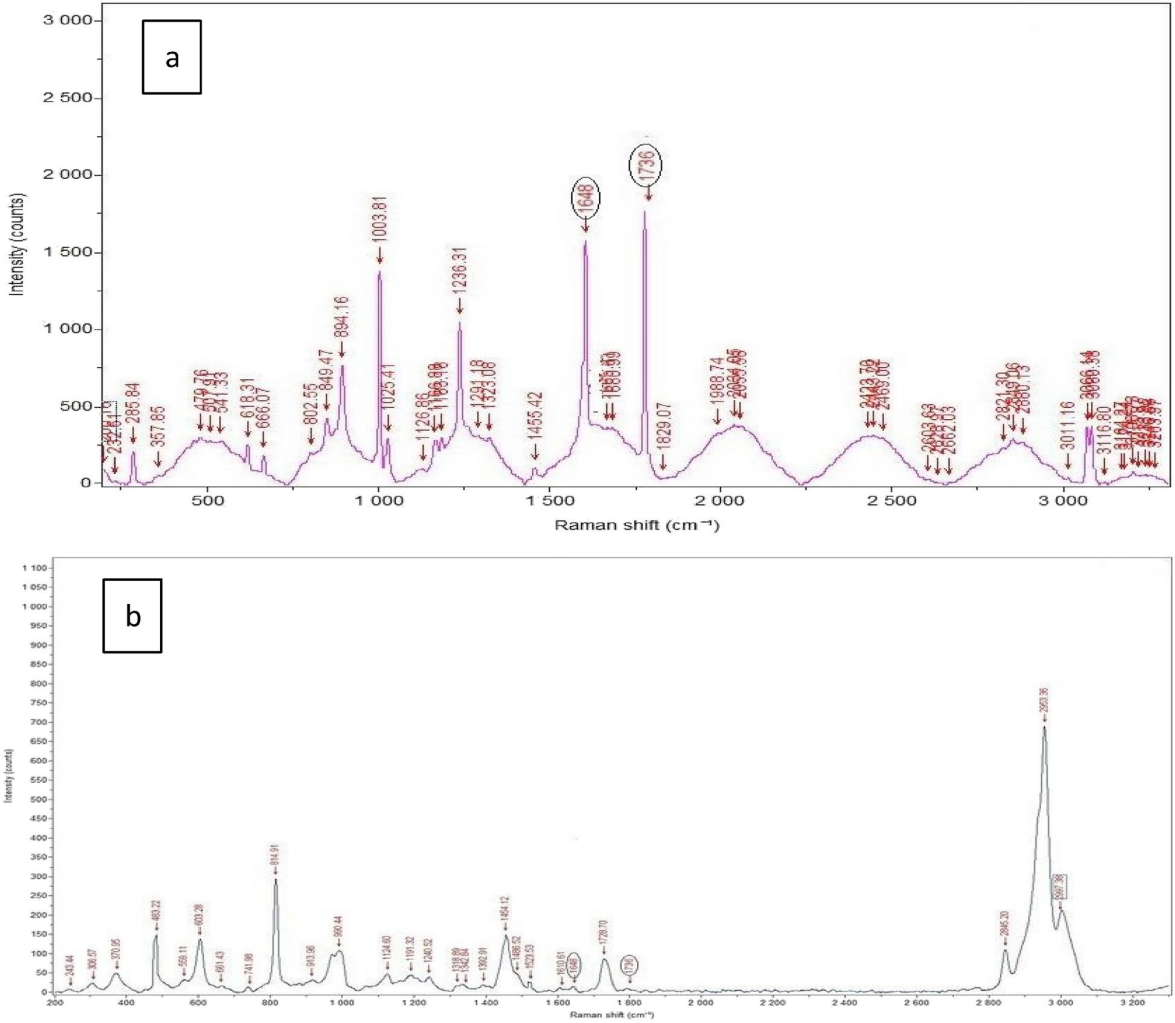
Raman chart for (**a**) methyl methacrylate monomer and (**b**) Poly methyl methacrylate. Changes in intensity of 1648 cm-1 and 1736 peaks between figure a and figure b mirrored the decrease in the number of remaining C = C double bonds during the polymerization that denote high degree of polymerization

The results showed that among the observed Raman bands of MMA, the bands at 1648 and 1736 cm^−1^ are attributed to (C = C) and (C═O) modes, respectively, and hence these two peaks can be used as an internal standard for qualitative determination of the degree of conversion. Changes in intensity of 1648 cm^−1^ and 1736 cm^−1^ peaks mirrored the decrease in the number of remaining C = C double bonds during the polymerization (Fig. 4). The reduction in the height of these two peaks denotes a very high degree of conversion.

### Results of scanning electron microscope

#### Scanning of the ceramic preform before polymer infiltration

SEM of PILN-25 and PILN-20 (Fig. 3) confirmed that the lithium disilicate ceramic particles were linked to each other, forming a ceramic network with variable-sized porosities. It was also observed that the porosity percentage was higher in PILN-25 than PILN-20.

#### Results of field emission scanning electron microscope

**Fig. 5:**
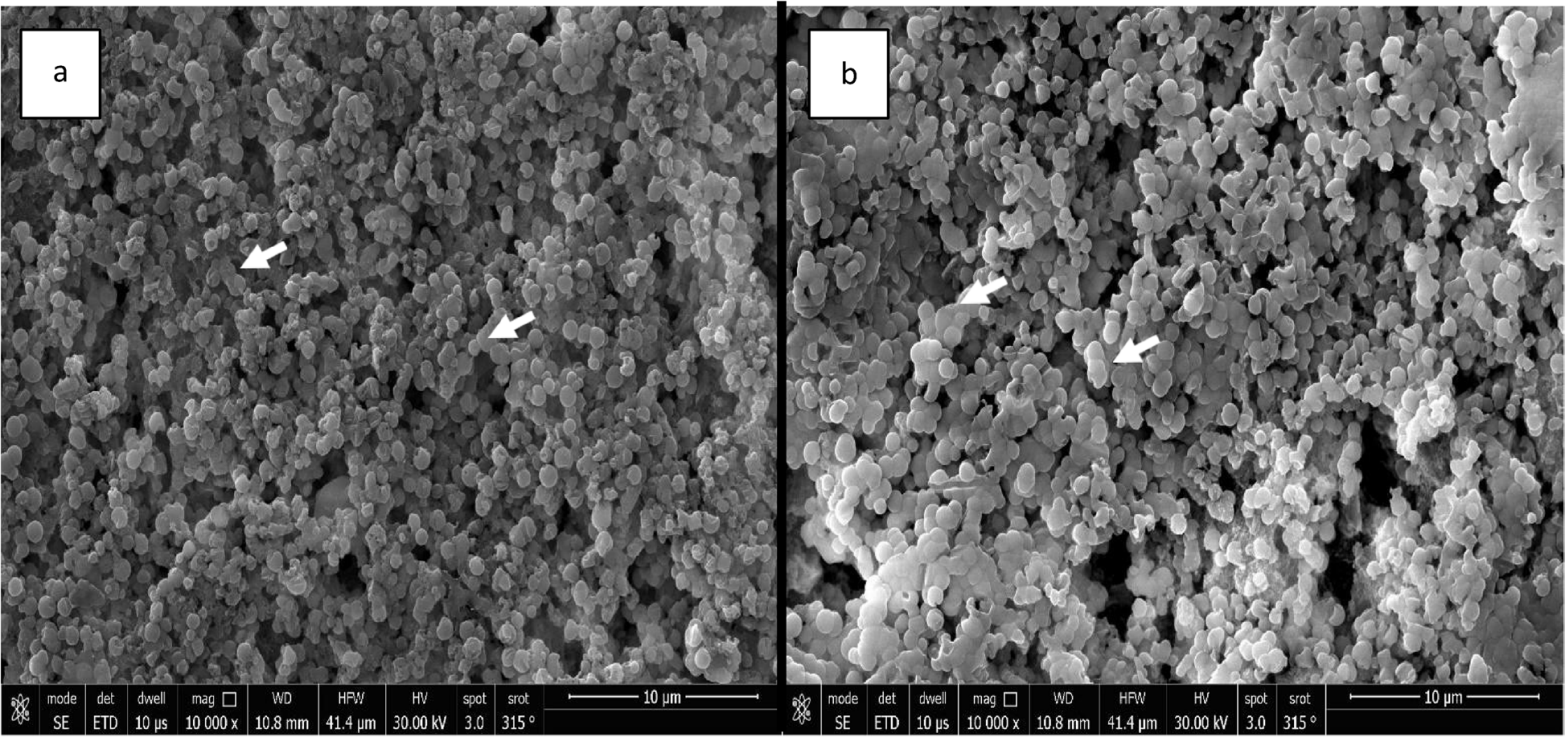
FE-SEM of ceramic preforms, both specimens revealed closely packed multi-directionally randomly oriented interlocking microstructure of numerous spherical-shaped lithium disilicate crystals dispersed throughout the specimens. **a** PILN-25 average particle size (~ 680 nm) with aspect ratio of (~ 0.75), While (**b**) PILN-20 average particle size (~ 710 nm) with aspect ratio of (~ 0.8). (White arrows point to necking that occur in lithium disilicate crystals). Magnification 10000X

FE-SEM of PILN-25 and PILN-20 revealed a closely packed, multi-directional, randomly oriented interlocking microstructure of numerous spherical-shaped lithium disilicate crystals dispersed throughout the specimens (Fig. 5). PILN-25 consisted of particles smaller than those of PILN-20, with an average particle size of (~ 680 nm) and an aspect ratio of (~ 0.75), compared to PILN-20 with a particle size of (~ 710 nm) and an aspect ratio of (~ 0.8) respectively. It was also noticed that some of the lithium disilicate crystals fused together, resulting in necking between particles.

#### Scanning of the ceramic preform after polymer infiltration

**Fig. 6:**
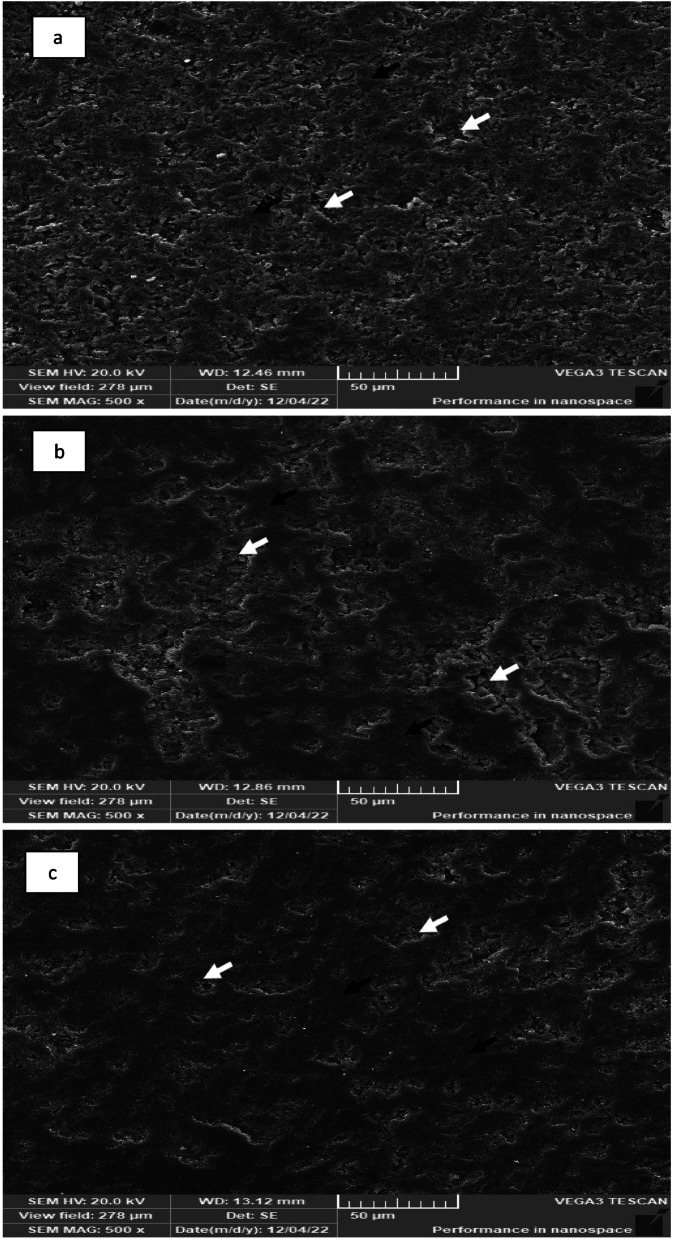
SEM showing the two interconnected networks (**a**) Enamic (**b**) PILN-25 (**c**) PILN-20 (white arrows point the polymeric network and black arrows point the ceramic network)

SEM of Enamic, PILN-25 and PILN-20 (Fig. 6) revealed a dense structure without apparent pores, in which a dominant ceramic network structure and polymeric network were fully merged.

#### Scanning after thermal treatment

**Fig. 7:**
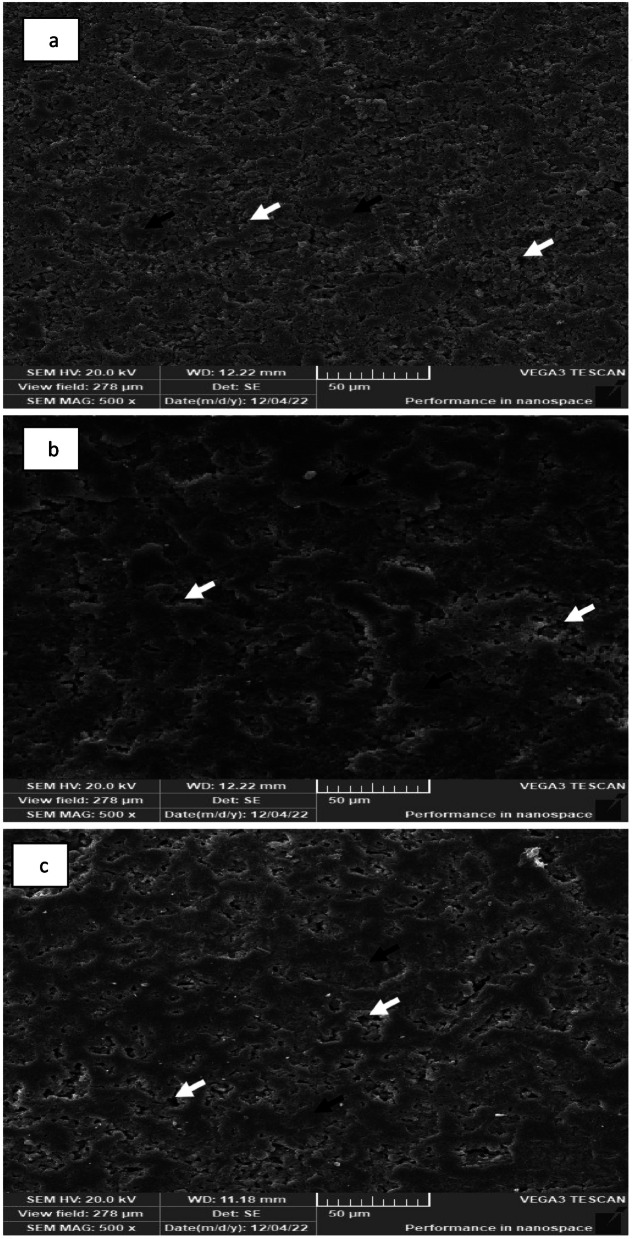
SEM of thermally etched different polymer infiltrated ceramic network (**a**) Enamic (**b**) PILN-25 and (**c**) PILN-20 (Black arrows point ceramic network while arrows point the porosity that was formerly occupied by polymeric network). Magnification 500X

SEM of Enamic, PILN-25 and PILN-20 (Fig. 7) showed a connected ceramic network with varying sizes of porosities, which were previously occupied by the polymeric networks. The porosities were scattered throughout the ceramic network.

#### Scanning after chemical treatment

**Fig. 8:**
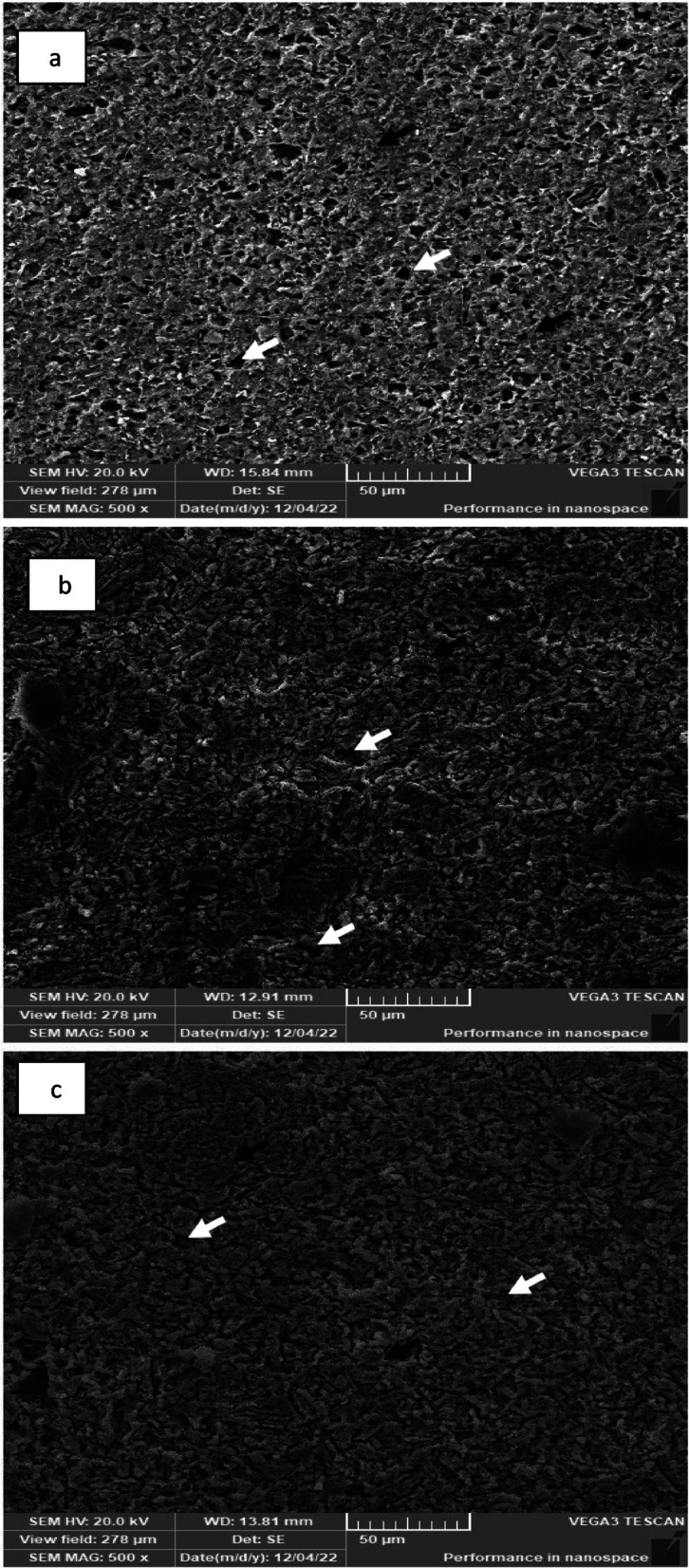
SEM of chemically etched different polymer infiltrated ceramic network (**a**) Enamic (**b**) PILN-25 and (**c**) PILN-20. (Black arrows point polymeric network while arrows point the porosity that was formerly occupied by ceramic network). Magnification 500X

SEM (Fig. 8) revealed that hydrofluoric acid selectively dissolves the surface ceramic component. For Enamic specimens, a honeycomb structure was observed, with porosity visible on the surface where the ceramic network was previously located. In contrast, PILN-25 and PILN- 20 displayed a sponge-like microstructure with numerous small pores of varying sizes scattered throughout the ceramic network.

### Biaxial flexural strength test

The results demonstrated that PILN-20 revealed the highest significant biaxial flexural strength (271 MPa), followed by PILN-25 (235.5 MPa), while Enamic revealed the lowest biaxial flexural strength (221.8 MPa) (Table 3).

**Table 3:** The mean, standard deviation (SD) and p-value of biaxial flexural strength MPa of the three groups: Enamic, PILN-25 and PILN-20 (*n* = 10)

Biaxial flexural strength test MPa						
Group	Enamic		PILN-25		PILN-20	
	Mean	SD	Mean	SD	Mean	SD
Biaxial flexural strength values	221.8^ ns^	45.31	235.5^ ns^	15.31	271^*^	35.73
*P* value	*P* < 0.05					

^*^Significant at *P* ≤ 0.05

*ns* non-significant

## Discussion

Efforts have been made to develop materials that not only tailor the mechanical properties necessary for the safe performance of the restoration but also achieve aesthetics that closely mimic natural teeth [27]. To date, several ceramic systems are available on dental market that can reproduce the teeth with great naturality [28] among which is the polymer infiltrated ceramic network. PICN, which has been strongly recommended as promising dental restorative material, was developed for CAD/CAM systems to emulate the properties of a natural tooth. Its dual interlocking phases combine rationally resulting in an elastic modulus of organic-inorganic composites similar to that of dentin [8].

While currently available PICN can mimic some of the mechanical properties of natural teeth, such as modules of elasticity and hardness, strength remains a challenge [29, 30]. Since the mechanical properties of the PICN greatly dependent on both phases (the type of the ceramic used, and the composition of the polymer applied) [9], fracture may be anticipated owing to the relative low strength of feldspathic ceramic present in the microstructure of the commercially available PICN [20]. Therefore, this in-Vitro study was conducted to evaluate the effect of replacement of feldspathic ceramic in PICN with lithium disilicate ceramics.

In the current study, the sintering parameters for porous lithium disilicate preform preparation were optimized. A two-stage heating schedules protocol was used, since this approach has been shown to yield higher mechanical properties due to the formation of smaller lithium disilicate crystals compared to a single-stage heating schedule [31–33]. In the first heating stage, the temperature increased at a ramp rate of 90 °C/min from room temperature to 550°C [34] and held at this temperature for 2 hours. This process provides healing and drying of the green body preform, combusts PVA, and removes any residual organic components [9]. In the second heating stage, the green body preforms were sintered for 1 hour at two different temperatures, 820 and 830 °C with a heating rate of 30 °C/min [33–36].

The preforms were then pretreated with silane coupling agent to ensure chemical bonding between the polymeric network and the ceramic network by forming a siloxane bond correspondingly to an increase in the surface energy of ceramic substrates and improve resin wettability. The infiltration of the preforms with silane coupling agent was achieved with a dip-coating process for 8 h following by heating at 60ºC in order to complete the silane-ceramic condensation reaction and to make the covalent bond more effective and resistant. Finally, the silanized preforms were immersed fully inside the monomer mixture of methyl methacrylate mixed with 0.5wt% benzoyl peroxide [9, 37].

The infiltration of MMA into the preforms was assisted by (1) ultrasonic force, (2) spin motive force, and (3) high centrifugal pressure. The applied pressure impacts the strength and the microstructure of PICN in two keyways. First, it results in a higher degree of conversion and crosslinking (Fig. 4b), leading to increased polymer strength. Second, the process pressure enables a higher monomer content in the performs by reducing the distance between monomer molecules, which leads to a higher amount of monomer and simultaneously of polymer in the PICN. This pressure-induced increase in monomer density reduces defects within PICN [38]. High temperature polymerization was then carried out in the water bath at 70 ◦C for 6 hours, with a heating rate of 2°C/min [39]. This method enhances the degree of conversion by increasing radical and monomer mobility, improving the flexibility of macromolecular chains, and allowing the remaining monomers to be incorporated into the chains [40–42]. The degree of conversion greatly influences resin characteristics such as flexural strength, dimensional stability, solubility, and degradation [43].

The porosity percentage of the specimens was measured using the helium pycnometer method, which is capable of measuring nanoscale open porosity, resulting in more precise results [44]. Among the two groups, PILN-20 exhibited a lower porosity percentage both before and after polymer infiltration stages as supported by SEM analysis (Fig. 3). This can be attributed to the direct relationship between porosity and firing temperature, with porosity decreasing as sintering temperature increases [9, 45, 46]. Porosity also reflects the strength and stability of ceramic materials [44]. At temperatures above the glass transition temperature, the viscosity of the glassy phase decreases, allowing softened glass to fill spaces between crystalline phases [45]. Due to the surface tension resulting from the softening of glassy material, pores become spherical and contract in volume. This explains why firing at 830 °C results in a lower porosity percentage [44]. This can explain why firing at (830 ^O^C) gives a lower porosity percentage.

The density of the material depends on several factors such as starting powder particle size. The density of ceramics tends to increase linearly with decreasing particle size of the starting powder. Using a fine starting powder with narrow size distribution can influence density since small particles have high surface energy. It has been reported that the decrease in surface energy is the driving force behind the densification process. Consequently, using fine particles can increase the strength of the ceramic product [45, 47–49]. Density also depends on sintering temperature and amount of porosity. As the sintering temperature increases, greater inter-particle contact occurs, resulting in subsequent bridge and neck formations between particles, which leads to ceramic material with less porosity and higher density [6, 50, 51]. This clarifies the results of the current study, as PILN-20 showed higher density than PILN-25 in both before and after infiltration stages.

Bulk fracture has been reported as the major cause of failure of CAD/CAM restorations [52], understanding the physical and mechanical properties of the materials may help to estimate clinical performance [53]. While no single property can be used to predict a material’s clinical success or failure, parameters such as flexural strength provide insight into the dynamic behavior of these materials under simulated occlusal stress [10]. Additionally, flexural strength characterizes the maximum stress level supported by the material under flexural stress, thus providing the most clinically relevant mechanical specification for brittle dental ceramics [51]. Flexural strength testing has long been a basic test to determine the resistance of ceramics to fracture, and high value is essential to successful clinical procedure [54, 55].

The results of the current study demonstrated that PILN-20 exhibited the highest significant biaxial flexural strength (271 MPa), followed by PILN-25 (235.5 MPa). This can be explained by the effect of firing temperature on the strength of ceramic material [51]. Sintering temperature can affect the strength of lithium disilicate ceramics in several ways, including its direct impact on porosity of ceramic material [45, 50]. Furthermore, the effect of reduced porosity is also reflected in density and, hence, strength. At high temperature, low porosity allows for greater inter-particle contact, which is followed by bridging between particle grains and neck formations, resulting in higher density. Stronger necks can disperse stress in many directions, leading to increased strength (Fig. 5) [6, 45, 51].

Another factor contributing to strength enhancement is the high aspect ratio of particles in PILN-20 (~0.8). Particles with high aspect ratio exhibit high mechanical properties. Since high aspect ratio denotes high homogeneity and regularity in the particle shape [56]. It also enhances the “interlocking effect” of crystalline phase [57]. Furthermore, high aspect ratio results in higher micro-residual stresses [31, 58], which are considered one of the key factors for strengthening lithium disilicate ceramics [59, 60]. The coefficient of thermal expansion mismatch between the glassy phase and the crystalline phase creates residual tensile stresses along the radial direction of the crystals. These residual tensile stresses are balanced by residual compressive stresses in the glass matrix around the crystal along the tangential direction. The generated residual compressive stresses in the glass matrix improve fracture resistance by promoting crack deflection [57, 61]. In contrast, particles with low aspect ratio displayed irregular shapes, which induce stress, raising flaws, and disrupt the interfacial interaction between the matrix and particles [56].

**Fig. 9:**
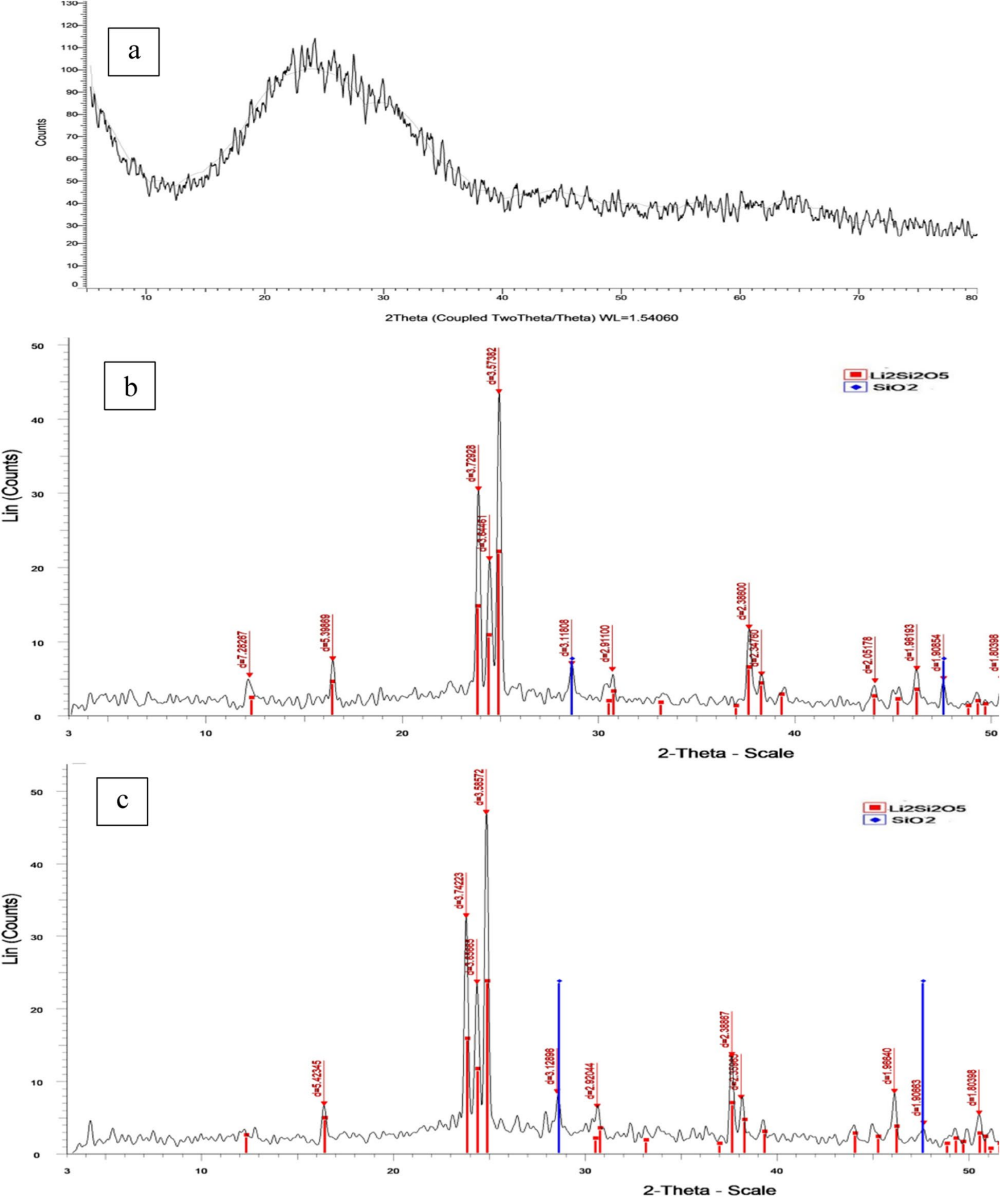
XRD patterns of (**a**) Enamic specimens displayed no dominant peaks, only a hump peak which indicates amorphous material without crystalline phases, (**b**) PILN-25 and (**c**) PILN-20 showed sharp well-defined peaks refer to cristobalite (blue lines) and lithium disilicate crystals (Li2Si2O5) (red lines)

Moreover, it has been reported that the strength of lithium disilicate ceramic is strongly affected by phase transformation, which is directly related to sintering temperature and firing cycles. The precursor lithium metasilicate (Li2SiO3) phase grows rapidly after nucleation and completely decomposes at 820 °C. Therefore, increasing the sintering temperature results in formation of the stronger lithium disilicate phase and decomposition of the weaker lithium metasilicate phase [51]. According to the results of ongoing study, PILN-20 was fired at a higher temperature (830 ^O^C) which contributed to its highest biaxial flexural strength due to the presence of a greater amount of lithium disilicate crystals. This is supported by the diffraction intensity of the crystalline phase from the XRD pattern (Fig. 9), which revealed that PILN-20 contains a higher amount of lithium disilicate.

Finally, Enamic revealed the lowest biaxial flexural strength (221.8 MPa). This can be explained by the fact that lithium disilicate, the type of ceramic used in fabrication of PILN in the current study, has higher mechanical properties than the feldspathic ceramic used in fabrication of commercially available PICN (Enamic).

## Conclusion

With the limitations of the current study, the following conclusions could be drawn:

1. Lithium disilicate based polymer infiltrated ceramic network resulted in higher biaxial flexural strength than feldspathic based polymer infiltrated ceramic network.
2. Sintering temperature has a high impact on the physical and mechanical properties of the material, as demonstrated by the higher strength of PILN-20 (fired at 830.^O^C)
3. Preparation of lithium disilicate based polymer infiltrated ceramic network may be a promising alternative to feldspathic based polymer infiltrated ceramic network as indirect ceramic restorative material.

### Limitation

Although urethane dimethacrylates are the most common choice for preparation of PICNs, polymethyl methacrylates were used in the current study due to supply chain issues during covid- 19 pandemic.

## Supplementary Information

Supplementary Material 1.

Supplementary Material 2.

## Data Availability

No datasets were generated or analysed during the current study.

## Abbreviations

CAD/CAM: Computer aided design/ computer aided manufacturing
PICN: Polymer infiltrated ceramic network
PILN: Polymer infiltrated lithium disilicate network
