# Supplementary material for: Physical and chemical characterization of experimental newly formulated polymer infiltrated lithium disilicate ceramic network versus polymer infiltrated feldspathic ceramic network (an in-vitro study)
Source: BMC Oral Health. 2025 Jun 5;25:918. doi: 10.1186/s12903-025-06134-8 (PMC12142892; doi:10.1186/s12903-025-06134-8)
Supplement: Supplementary file 2 — Supplementary Material 2. [file 12903_2025_6134_MOESM2_ESM.pdf]

Pilot study

1. Selection of firing temperature

Different temperatures were chosen starting from 800 ^{O}C to ensure transformation of all lithium metasilicate crystals to lithium disilicate. The green body preforms were sintered in furnace without vacuum following two stage heat treatment protocol of lithium disilicate ceramics: Firstly, the samples were fired at 600 °C. Then, the samples were sintered for 2 h at seven different temperatures (800, 810 820, 830, 840, 850 and 900 °C). Sample 900 was excluded as the high temperature resulted in deformation of the green body preform. While other samples were subjected to porosity testing to choose optimum porosity percentage to be used in the study. After sintering, the apparent porosity of the samples was measured using two methods fluid saturation method and gas expansion method. The results are presented in table 1

Table 1: The results of porosity % using fluid saturation and gas expansion methods in relation to different sintering temperature.

Firing temperature		800	810	820	830	840	850
Porosity	Fluid saturation	24.39%	23.49%	13.41%	6.53%	2.21%	1.12%
	Gas expansion	32.20%	28.19%	24.95%	19.71%	5.21%	1.88%

Sample 800 and 810 were excluded from the study due to high porosity ratio, while sample 840 and 850 were excluded from the study due to low porosity ratio.
